# Performance of recurrent neural networks with Monte Carlo dropout for predicting pharmacokinetic parameters from dynamic contrast‐enhanced magnetic resonance imaging data

**DOI:** 10.1002/acm2.14586

**Published:** 2024-12-23

**Authors:** Kenya Murase, Atsushi Nakamoto, Noriyuki Tomiyama

**Affiliations:** ^1^ Department of Future Diagnostic Radiology Graduate School of Medicine Osaka University Suita Osaka Japan; ^2^ Department of Diagnostic and Interventional Radiology Graduate School of Medicine Osaka University Suita Osaka Japan

**Keywords:** dynamic contrast‐enhanced MRI, gated recurrent units (GRU), long short‐term memory (LSTM), Monte Carlo dropout, pharmacokinetic parameters, recurrent neural networks

## Abstract

**Purpose:**

To quantitatively evaluate the performance of two types of recurrent neural networks (RNNs), long short‐term memory (LSTM) and gated recurrent units (GRU), using Monte Carlo dropout (MCD) to predict pharmacokinetic (PK) parameters from dynamic contrast‐enhanced magnetic resonance imaging (DCE‐MRI) data.

**Methods:**

DCE‐MRI data for simulation studies were synthesized using the extended Tofts model and a population‐averaged arterial input function (AIF). The ranges of PK parameters for training the RNNs were determined from data of patients with brain tumors. The effects of the number of training samples, number of hidden units, dropout rate (DR), and bolus arrival time delay and dispersion in AIF on the accuracy of the PK parameters were investigated, and the uncertainties for different DRs and peak signal‐to‐noise ratios (PSNRs) were quantified. For comparison, PK parameters were estimated using the nonlinear least‐squares method. In the clinical studies, the PK parameter and uncertainty images were generated by applying the trained RNNs to DCE‐MRI data.

**Results:**

Compared with GRU, the computational cost for training the LSTM was significantly higher. The prediction accuracy of GRU decreased with decreasing numbers of training samples and hidden units, whereas the performance of LSTM remained stable. Despite an increased computational cost, MCD reduced the prediction error at low PSNR and improved the quality of PK parameter images. The simulation results recommended using a DR of 0.25–0.5 at low PSNR and ≤ 0.25 for other PSNRs. The clinical studies recommended using a DR of 0.25 and 0.5 for LSTM and GRU, respectively.

**Conclusions:**

MCD is effective in quantifying uncertainty in PK parameter prediction from DCE‐MRI data and improves their performance, particularly at low PSNR; however, at the expense of increased computational cost. This study helps deepen our understanding of RNNs with MCD and select suitable hyperparameters for creating an RNN architecture for DCE‐MRI studies.

## 1 INTRODUCTION

Dynamic contrast‐enhanced magnetic resonance imaging (DCE‐MRI), performed after intravenous administration of a contrast agent (CA), is a promising tool for the non‐invasive investigation of vascular characteristics in tumors^1^ and aids in the quantification of pharmacokinetic (PK) parameters. Assessing the pathophysiological state of tumors using these PK parameters is important for determining medical treatment plans and/or predicting prognosis in patients with tumors.^2^, ^3^ One of the advantages of using DCE‐MRI is that, unlike DCE‐CT, the input functions required for PK parameter quantification can be obtained noninvasively and without radiation exposure.

Conventionally, PK parameters have been estimated by fitting a tracer kinetic model, such as the extended Tofts model^4^ to observed data using the nonlinear^5^ or linear least‐squares method.^6^ However, recent advancements in artificial intelligence (AI) have enabled the exploration of convolutional neural networks (CNNs)^7^, ^8^ and recurrent neural networks (RNNs)^9^, ^10^ for predicting PK parameters directly from DCE‐MRI data. However, Ottens et al.^10^ reported that CNN‐based methods are not well suited for processing time‐series data, and the models can only be trained on the data‐specific total acquisition length. In contrast, RNNs are designed for learning sequential data and have been applied to various fields, including time‐series prediction, such as natural language processing and speech recognition. Therefore, RNN‐based methods can handle data with arbitrary input and output lengths.

Two types of advanced RNNs, namely the long short‐term memory (LSTM)^11^ and gated recurrent units (GRU)^12^, ^13^ have been designed to address the vanishing gradient problem in traditional RNNs. The details of these network architectures have been previously described.^13^ Briefly, the LSTM consists of three gates (input, forget, and output) that regulate the flow of information into and out of each unit of the network. The GRU is a simplified version of the LSTM with only two gates (reset and update). These gates determine how much of the previous state should be reset and how much of the new input should be used to update the state at each unit of the network. These gating mechanisms enable RNNs to effectively retain important information and discard irrelevant information over long sequences, making them suitable for complex tasks.

The PK parameters predicted using these approaches include uncertainties derived from inherent noise in the training data (aleatoric uncertainty) and a lack of knowledge about the model parameters or a mismatch between the data used in training and testing (model or epistemic uncertainty). The predictive uncertainty represents the variation in predicted values caused by these factors and is a measure of how likely we can trust the predicted values. Quantification of the predictive uncertainties of the PK parameters is important for evaluating their accuracy and reliability. Bayesian neural networks have been proposed for uncertainty quantification.^14^, ^15^ These models incorporate prior probability distributions with network, and estimate predictive uncertainties from posterior probability distributions.^14^, ^15^ Although effective Bayesian neural networks incur substantial computational costs for estimating predictive uncertainties when applied to large datasets. Monte Carlo dropout (MCD) is another method for quantifying uncertainties in neural networks.^16^, ^17^ The MCD is a regularization method for preventing overfitting in neural networks that randomly deactivate nodes or neurons in neural networks during training. MCD is not usually applied in testing, while the network weights are adjusted by considering the dropout rate (DR), leading to deterministic predictions. However, when MCD is applied both during training and testing, the prediction is no longer deterministic. By running the trained neural networks multiple times with the same inputs, the outputs are probabilistic, and the mean of the probability distribution can be used as the predicted value and the variance as a measure of its uncertainty.^17^ Gal et al.^17^ theoretically demonstrated that MCD can be interpreted as an approximate Bayesian inference. The MCD method is much simpler to implement than Bayesian approaches and can estimate predictive uncertainties with minor modifications to the existing model architecture.

This study aimed to evaluate the performance of LSTM and GRU incorporated with MCD for predicting PK parameters from DCE‐MRI data and to evaluate their usefulness in comparison with the nonlinear least‐squares (NLSQ) method through simulation and clinical studies.

## 2 MATERIALS AND METHODS

### 2.1 Recurrent neural networks

**FIGURE 1:**
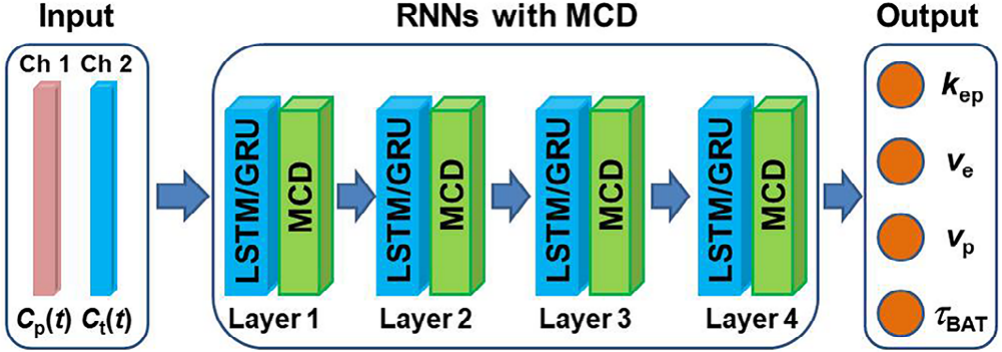
Illustration of the RNN architecture used in this study for predicting PK parameters. The input of the RNNs comprises two channels: the first and second channels are the time‐dependent concentration of contrast agent in the plasma of the feeding artery (*C_p_* (*t*)) and tissue (*C_t_* (*t*)), respectively. The output comprises four PK parameters (*k*_ep_, *v*_e_, *v*_p_, and *τ*_BAT_). GRU: gated recurrent units; LSTM, long short‐term memory; MCD: Monte Carlo dropout; PK, pharmacokinetic; RNN, recurrent neural network.

Figure 1 illustrates the RNN architecture used in this study, which consists of the input, RNNs (LSTM or GRU) incorporated with MCD, and output. The input of the RNNs comprises two channels: the first channel represents the time‐dependent concentration of CA in the plasma of the feeding artery (*C_p_* (*t*)), and the second channel represents the concentration in the tissue (*C_t_* (*t*)). The output comprises four PK parameters (*k*_ep_, *v*_e_, *v*_p_, and *τ*_BAT_). The RNNs were configured with four hidden layers. Batch normalization was used after each RNN layer except for the last layer, where a fully connected (dense) layer with four output parameters was used instead of batch normalization. When the MCD and the first channel of input are not used, our RNN architecture is basically the same as DCE‐NET (https://github.com/oliverchampion/DCENET). The DCE‐NET ranks first among the 10 challenge entries in OSIPI (Open Science Initiative for Perfusion Imaging) in terms of accuracy, repeatability, and reproducibility.^18^

In this study, the DRs were set to 0, 0.25, and 0.5. The LSTM models with DRs of 0, 0.25, and 0.5 are denoted as LSTM(0), LSTM(0.25), and LSTM(0.5), respectively. Similarly, the GRU with DRs of 0, 0.25, and 0.5 are referred to as GRU(0), GRU(0.25), and GRU(0.5), respectively.

### 2.2 Simulation study

#### 2.2.1 Generation of synthetic data using extended Tofts model

In the extended Tofts model,^4^ $C_{t}\left(t\right)$ is expressed as^10^

(1) $$\begin{array}{ccc} C_{t}\left(t\right) & = & k_{\mathrm{ep}}\;\cdot v_{e}\cdot \underset{0}{\int^{t}}C_{p}\left(\tau -\tau_{\mathrm{BAT}}\right)\\ & & \times \;e^{-k_{\mathrm{ep}}\left(t-\tau \right)}d\tau +v_{p}\cdot C_{p}\left(t-\tau_{\mathrm{BAT}}\right) \end{array}$$

where *k*_ep_, *v*_e_, and *v*_p_ denote the transfer constant from the extravascular extracellular space to the blood plasma, volume of extravascular extracellular space per unit volume of tissue, and blood plasma volume fraction, respectively. In Equation (1), $\tau_{\mathrm{BAT}}$ denotes the bolus arrival time (BAT) of the CA at the tissue region of interest. When deriving Equation (1), $C_{p}\left(t\right)$ was assumed to be zero at $t\leq \tau_{\mathrm{BAT}}$. Considering the dispersion effect on *C_p_* (*t*) and hematocrit (Hct) in blood, *C_p_* (*t*) is expressed as

(2) $$C_{p}\left(t\right)=\frac{\mathrm{SF}}{1-\mathrm{Hct}}\;\cdot \underset{0}{\int^{t}}C_{b}\left(\tau \right)\cdot \frac{1}{\tau_{d}}e^{-\left(t-\tau \right)/\tau_{d}}d\tau .$$

where $\tau_{d}$ denotes the dispersion constant and *C_b_* (*t*) is the CA concentration in the arterial blood at time *t*, that is. arterial input function (AIF). SF denotes a scaling factor that considers the effects of the injected dose and Hct variations. In this study, the population‐averaged AIF^19^ was used as $C_{b}\left(t\right)$ and converted to $C_{p}\left(t\right)$ using Equation (2), with Hct assumed to be 0.45. The time‐concentration curves (*C_p_* (*t*) and *C_t_* (*t*)) comprised 65 frames with a temporal resolution of 4.8 s per frame.

The volume transfer constant from blood plasma to the extravascular extracellular space (*K* ^trans^) was calculated using *k*_ep_ and *v*_e_ as follows:^4^

(3) $$K^{\mathrm{trans}}=k_{\mathrm{ep}}\;\times v_{e}.$$

To simulate the DCE‐MRI studies of patients with brain tumors, *k*_ep_, *v*_e_, and *v*_p_ in Equation (1) were randomly selected in the following ranges: 0.00025–0.33 min^−1^, 0.04–0.6, and 0.005–0.1, respectively, as described by Fang et al.^20^ Furthermore, *τ*_BAT_ was randomly selected in the range of 40–50 s based on clinical data.^9^ The SF in Equation (2) was randomly selected in the range between 0.7 and 1.3,^9^ and $\tau_{d}$ was randomly selected in the range of 0.1–4 s based on the data reported by Calamante et al.^21^ These parameters used in training and testing were generated from uniform random numbers distributed within the corresponding ranges that equal to the number of the training and test samples, respectively.

To simulate statistical noise, Gaussian noise was added to $C_{t}\left(t\right)$. The noise was randomly sampled from a Gaussian distribution with zero mean and standard deviation (SD) given by $\mathrm{SD}=\max(C_{t}\left(t\right))/\mathrm{PSNR}$, where $\max(C_{t}\left(t\right))$ and PSNR denotes the maximum value of $C_{t}\left(t\right)$ and the peak signal‐to‐noise ratio (PSNR), respectively. The PSNR was assumed to be distributed between 20 and 30 in the training samples.^9^ The cases with PSNRs of 10, 15, and 20 to 30 were considered for the test samples. Unless otherwise noted, the PSNR of 20–30 was used. Specifically, when considering the PSNR distributed between 20 and 30 in training and testing, the PSNRs equal to the number of the training and test samples were distributed into this range using a geometric progression, respectively. For each PSNR and sample, Gaussian noises equal to the number of frames (65 in this study) were generated from normally distributed random numbers with zero mean and SD of $\max(C_{t}\left(t\right))/\mathrm{PSNR}$.

When training and testing the RNNs, the $C_{p}\left(t\right)$ with no BAT delay and dispersion and SF of unity was used as the first channel and the $C_{t}\left(t\right)$ generated from Equations (1) and (2) was used as the second channel of input in the RNNs (Figure 1).

#### 2.2.2 Training and validation of recurrent neural networks

The ratio of the training and validation datasets was taken as 0.9/0.1. The batch size and the total number of epochs were fixed at 128 and 50, respectively. The network was optimized by minimizing the loss function (mean squared error between the predicted PK parameters and corresponding ground truth values) using the Adam optimizer^22^ with an initial learning rate of 10^−3^ and a decay constant of 0.1. Unless otherwise noted, the numbers of training samples and hidden units were set to 10^5^ and 256, respectively. The training and validation of the RNNs were repeated five times for each training configuration.

The RNNs were implemented in Python 3.10.12 using Keras 3.5.0 on a Tensorflow 2.17.1 backend. The training, validation, and testing were performed using an NVIDIA Tesla T4 graphics processing unit (GPU) on the Google Cloud Platform.

#### 2.2.3 Prediction and uncertainty quantification of pharmacokinetic parameters and evaluation

The predicted value and uncertainty for each PK parameter were calculated by running the trained RNNs 100 times using the same input (test) data. The mean of the output data for *n* = 100 was used as the predicted value, and the SD was used as the uncertainty for each PK parameter. In this study, the uncertainty ($\sigma_{uc}$) of the PK parameter *θ* (*θ*: *k*_ep_, *v*_e_, *v*_p_, *K* ^trans^, and *τ*_BAT_) was normalized by the ground truth value ($\theta_{gt}$) range and expressed in percent as follows:

(4) $$\sigma_{uc}=\frac{\mathrm{SD}\left(\theta \right)}{\max\left(\theta_{gt}\right)-\min\left(\theta_{gt}\right)}\;\times 100\;\left(\%\right),$$

where SD(θ) denotes the SD of *θ* for *n *= 100 and $\max\left(\theta_{gt}\right)\;\mathrm{and}\;\min\left(\theta_{gt}\right)$ denote the maximum and minimum values of $\theta_{gt}$, respectively.

The above predictions were performed using the same RNNs with 1000 different input (test) data. The predicted values (1000 for each PK parameter) were used to calculate the concordance correlation coefficient (CCC)^23^ and the normalized root mean square error (NRMSE) to quantitatively evaluate the accuracy of the predicted PK parameters. The mean of $\sigma_{uc}$ (Equation 4) for *n* = 1000 was used for statistical analysis.

The CCC (*r*_ccc_) measures the agreement between two variables, and its relation to the ordinary Pearson's correlation coefficient (*r*_cc_) is expressed as^23^

(5) $$r_{\mathrm{ccc}}=\frac{2r_{\mathrm{cc}}\sigma_{x}\sigma_{y}}{\sigma_{x}^{2}+\sigma_{y}^{2}+{\left(\mu_{x}-\mu_{y}\right)}^{2}}\;,$$

where $\sigma_{x}^{2}$ and $\sigma_{y}^{2}$ denote the variances for the two variables *x* and *y*, and $\mu_{x}$ and $\mu_{y}$ are the corresponding mean values.

The NRMSE for θ was calculated as

(6) $$\mathrm{NRMSE}=\frac{\sqrt{\frac{1}{N_{t}}\sum_{i=1}^{N_{t}}{\left[\theta \left(i\right)-\theta_{gt}\left(i\right)\right]}^{2}}}{\max\left(\theta_{gt}\right)-\min\left(\theta_{gt}\right)}\;\times 100\;\left(\%\right),$$

where θ(i) and $\theta_{gt}\left(i\right)$ denote the predicted and ground truth values of θ for the *i*th test sample, respectively, and *N_t_* denotes the total number of test samples (1000 in this study).

For comparison, the same 1000 test samples were analyzed using the NLSQ method with constrained optimization (Python scipy.optimize curve_fit) to estimate PK parameters from Equations (1) and (2). When using NLSQ, the initial estimates for *k*_ep_, *v*_e_, *v*_p_, and *τ*_BAT_ were set to 0.15 min^−1^, 0.3, 0.03, and 45 s, respectively, and the corresponding bounds were set to [10^−6^, 0.5], [10^−8^, 1.0], [10^−8^, 0.1], and [10^−3^, 60], respectively. To account for BAT delay, *τ*_BAT_ was incorporated into Equation (1) for NLSQ estimation, whereas the dispersion effect was not considered in Equation (2), and the SF was assumed to be unity.

#### 2.2.4 Statistical analysis

For statistical analysis, the calculations of $\sigma_{uc}$ (Equation 4), CCC (Equation 5), and NRMSE (Equation 6) were repeated five times using different trained RNNs and input (test) data. When NLSQ was used, the CCC and NRMSE were calculated in the same manner. The $\sigma_{uc}$, CCC, and NRMSE values were represented as the mean ± SD for *n* = 5. Differences in these values among the groups were analyzed using one‐way analysis of variance. Statistical significance was determined using Tukey's multiple comparison test, and *p *< 0.05 was considered statistically significant. When analyzing the statistical differences in CCC, the CCC values were transformed to *z*‐values using Fisher's *z*‐transformation, that is, $z=\mathrm{lo}g_{e}\left[\left(1+r_{\mathrm{ccc}}\right)/\left(1-r_{\mathrm{ccc}}\right)\right]/2$, to improve the approximation of the normal distribution.^23^

When analyzing the statistical difference between the means of the two groups, Welch's *t*‐test was used, and *p *< 0.05 was considered statistically significant.

### 2.3 Clinical study

#### 2.3.1 Data processing

We used publicly available open clinical challenge data from the OSIPI repository,^18^ “https://github.com/OSIPI/TF6.2_DCE‐DSC‐MRI_Challenges,” and analyzed the DCE‐MRI data from eight patients with brain tumors (one slice including the brain tumor per patient). The details of the data acquisition protocol are described by Shalom et al.^18^ In this study, a spoiled gradient‐echo imaging sequence was assumed, and the concentration of CA at time *t* (*C*(*t*)) was calculated from the MRI signal intensity as follows: First, the pre‐contrast longitudinal relaxation time (*T*_10_) and MRI signal intensity (*S*_0_) were measured using the variable flip angle method.^24^ When using the variable flip angle method, the MRI signal intensity (*S*(*α*_VFA_)) at a flip angle of *α*_VFA_ is expressed as

(7) $$S\left(\alpha_{\mathrm{VFA}}\right)=K\cdot \frac{\left(1-E_{\mathrm{VFA}}\right)\cdot \sin\left(\alpha_{\mathrm{VFA}}\right)}{1-E_{\mathrm{VFA}}\cdot \cos\left(\alpha_{\mathrm{VFA}}\right)},$$

where *K* denotes the proportionality constant incorporating the proton density, coil sensitivity, and *T*_2_^*^ relaxation, and $E_{\mathrm{VFA}}=\exp\left(-TR_{\mathrm{VFA}}/T_{10}\right)$ where TR_VFA_ is the repetition time for MRI data acquisition using the variable flip angle method. In this study, $TR_{\mathrm{VFA}}$ was set at 4.43 ms and $\alpha_{\mathrm{VFA}}$ was varied as 5°, 10°, 15°, 20°, 25°, and 30°. The *K* and *T*_10_ values were calculated from Equation (7) using the NLSQ method, with their initial estimates obtained using the linear least‐squares method proposed by Fram et al.,^25^ and were used to calculate *S*_0_ from Equation (7). The *T*_10_ and *S*_0_ images were generated by applying these procedures pixel‐by‐pixel.

The reciprocal of the longitudinal relaxation time at time *t* after the CA injection (*T*_1_(*t*)) is expressed as

(8) $$\begin{array}{ccc} & & \frac{1}{T_{1}\left(t\right)}=-\frac{1}{TR_{\mathrm{DCE}}}\\ & & \;\times \;\mathrm{lo}g_{e}\left\{\frac{1-A\left(t\right)\cdot \left(1-E_{\mathrm{DCE}}\right)-E_{\mathrm{DCE}}\cdot \cos\left(\alpha_{\mathrm{DCE}}\right)}{1-\left[E_{\mathrm{DCE}}+A\left(t\right)\cdot \left(1-E_{\mathrm{DCE}}\right)\right]\cdot \cos\left(\alpha_{\mathrm{DCE}}\right)}\right\},\; \end{array}$$

where $E_{\mathrm{DCE}}=\exp\left(-TR_{\mathrm{DCE}}/T_{10}\right)$ and $A\left(t\right)=S\left(t\right)/S_{0}$ with *S*(*t*) being the MRI signal intensity at time *t* after CA injection. $TR_{\mathrm{DCE}}$ and $\alpha_{\mathrm{DCE}}$ denote the repetition time and flip angle for DCE‐MRI data acquisition, respectively. In this study, $TR_{\mathrm{DCE}}$ and $\alpha_{\mathrm{DCE}}$ were 3.8 ms and 25°, respectively.^18^ *S*_0_ was calculated by substituting the previously determined *K* and *T*_10_ values into Equation (7) and replacing $TR_{\mathrm{VFA}}$ and $\alpha_{\mathrm{VFA}}$ by $TR_{\mathrm{DCE}}$ and $\alpha_{\mathrm{DCE}}$, respectively. Finally, *C*(*t*) was calculated as follows:

(9) $$C\left(t\right)=\frac{1}{r_{1}}\;\cdot \left(\frac{1}{T_{1}\left(t\right)}-\frac{1}{T_{10}}\right),$$

where *r*_1_ is the longitudinal relaxivity of the CA. In this study, *r*_1_ was assumed to be 3.9 mM^−1^ s^−1^, which is the mean *r*_1_ value of gadolinium‐labeled diethylenetriaminepentaacetic acid (Gd‐DTPA) in water, blood, and plasma at 1.5 T.^26^

To calibrate *C*(*t*), it was multiplied by the calibration factor (CF), defined as

(10) $$\mathrm{CF}=\frac{\mathrm{AUC}\left(\mathrm{AIF}\right)}{\mathrm{AUC}\left(\mathrm{VOF}\right)}\;,$$

where AUC(AIF) and AUC(VOF) represent the areas under the curve of AIF (*C*_b_(*t*) in Equation 2) and the venous output function, respectively. In this study, the population‐averaged AIF^19^ was used as the AIF, and the venous output function was obtained by drawing the region of interest on the superior sagittal sinus to avoid the partial volume effect.^27^

Brain regions were extracted from the *S*_0_ images using the “make binary” function in ImageJ (http://rsb.info.nih.gov/ij/) and then manually corrected. To compare the CCC values within the tumor, tumor regions were extracted from the *K* ^trans^ images following the same procedure used for brain regions.

Similar to the simulation study, the trained RNNs with the same input data were run 100 times, and the predicted value and uncertainty for each PK parameter were calculated from the mean and SD of the 100 output values, respectively. These calculations were performed pixel‐wise within the brain region to generate PK parameters and uncertainty images.

#### 2.3.2 Statistical analysis

To compare the CCC values of the tumors among the groups, they were transformed into z‐values using Fisher's z‐transformation. Statistical differences in the transformed *z*‐values among groups were analyzed using a one‐way analysis of variance and Tukey's multiple comparison tests, with *p* < 0.05 indicating statistical significance.

The statistical difference in the predictive uncertainty between the two groups was analyzed using Welch's *t‐*test, and *p *< 0.05 was considered statistically significant.

## 3 RESULTS

### 3.1 Simulation study

#### 3.1.1 Relationship between loss and epoch number

**FIGURE 2:**
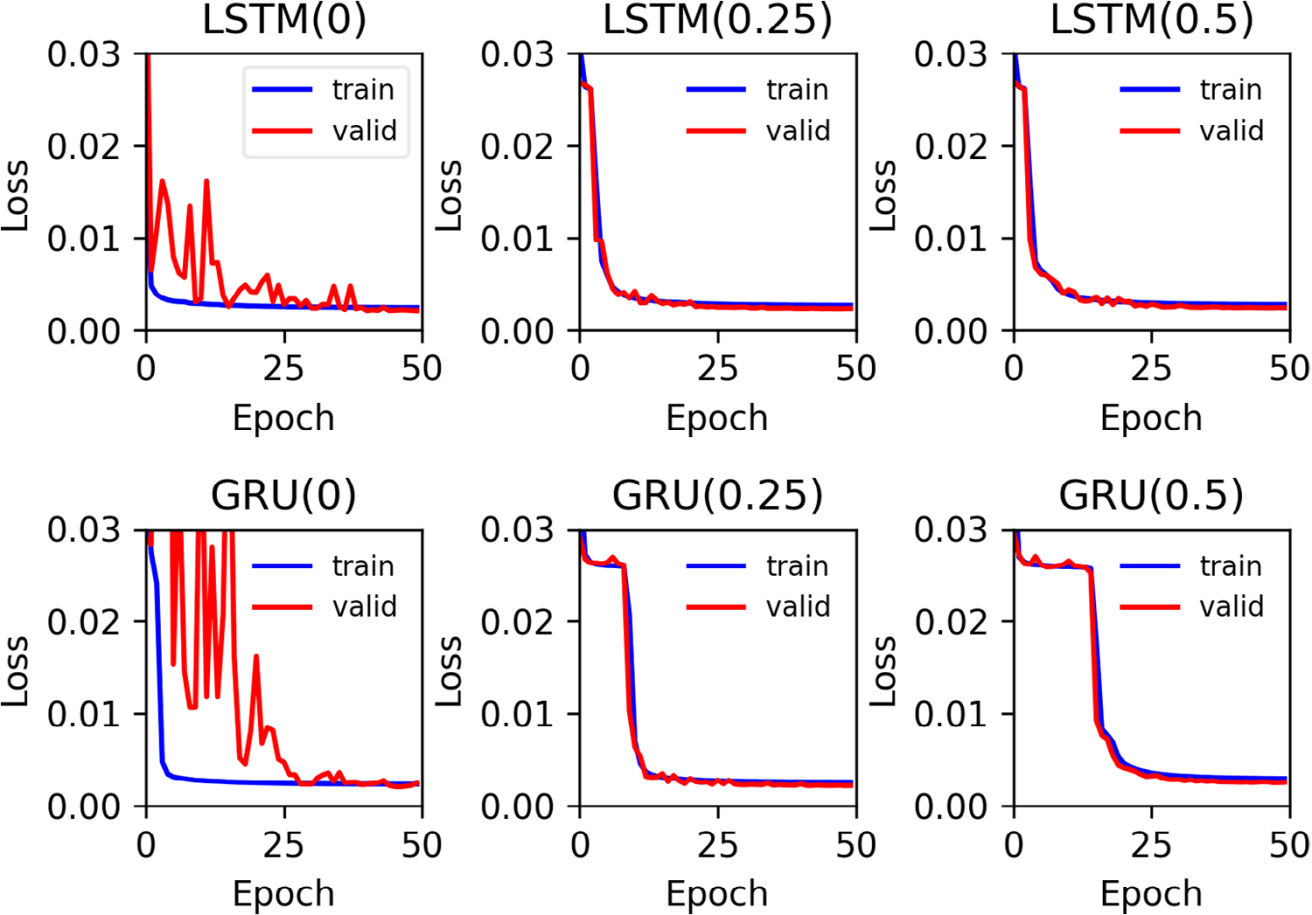
Training (blue solid lines) and validation losses (red solid lines) as a function of the epoch number for LSTM (upper row) and GRU (lower row) with DRs of 0, 0.25, and 0.5 (left to right column). DRs, dropout rates; GRU: gated recurrent units; LSTM, long short‐term memory.

Figure 2 shows examples of the training and validation losses as a function of the epoch number for LSTM (upper row) and GRU (lower row) with varying DRs. The left, middle, and right columns show the results for DRs of 0, 0.25, and 0.5, respectively. When the DR was zero, large differences between the training and validation losses were observed for epoch numbers less than approximately 40, and they disappeared with increasing epoch numbers thereafter in both LSTM and GRU. However, DRs of 0.25 or 0.5 effectively mitigated these differences even when the number of epochs was small.

#### 3.1.2 Performance of recurrent neural networks across varying number of training samples

**FIGURE 3:**
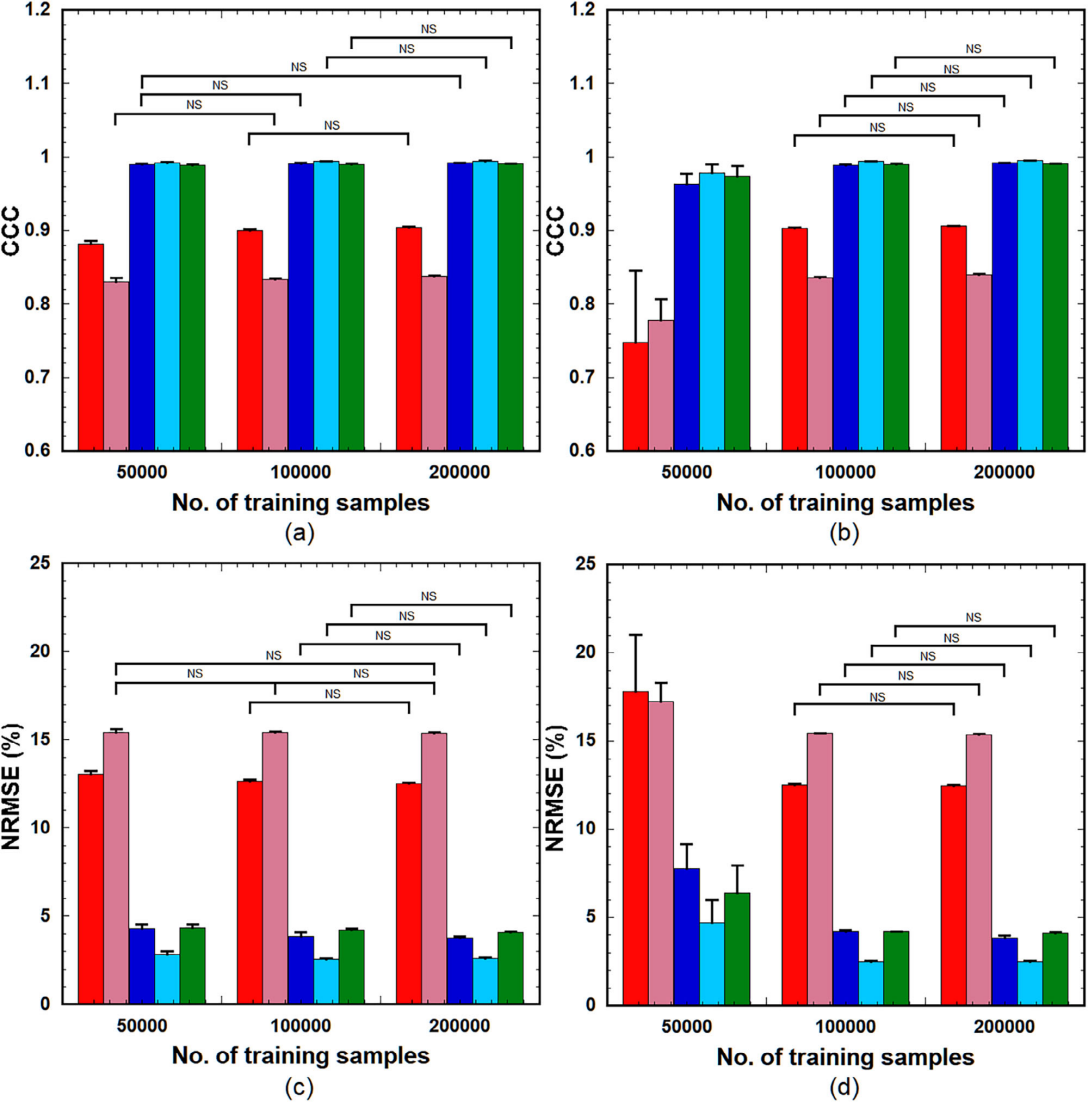
CCC (upper row) and NRMSE) (lower row) of the PK parameters predicted using LSTM(0.25) (left column) and GRU(0.25) (right column) across varying number of training samples (50000, 100000, and 200000) (left to right group). Red bar: *k*_ep_, pink bar: *v*_e_, blue bar: *v*_p_, light blue bar: *K* ^trans^, and green bar: *τ*_BAT_. Bar and error bar represent the mean and SD for *n* = 5, respectively. Significant differences were observed among parameters, excluding those indicated by ”NS” for each PK parameter. CCC, concordance correlation coefficient; GRU: gated recurrent units; LSTM, long short‐term memory; NRMSE, normalized root mean square error; PK, pharmacokinetic; NS, not significant.

Figure 3 shows the CCC (Equation 5) (upper row) and NRMSE (Equation 6) (lower row) of the PK parameters predicted using LSTM(0.25) (left panels) and GRU(0.25) (right panels) across varying training sample sizes (5 × 10^4^, 1 × 10^5^, and 2 × 10^5^). When using LSTM(0.25), significant differences in the CCC values were observed between 5 × 10^4^ and 1 × 10^5^ samples and between 5 × 10^4^ and 2 × 10^5^ samples for *k*_ep_, *K* ^trans^, and *τ*_BAT_, and between 5 × 10^4^ and 2 × 10^5^ samples for *v*_e_ and *v*_p._ No significant differences were observed in other combinations (Figure 3a). NRMSE exhibited significant differences for *k*_ep_, *v*_p_, *K* ^trans^, and *τ*_BAT_ between 5 × 10^4^ and 1 × 10^5^ and between 5 × 10^4^ and 2 × 10^5^ samples (Figure 3c). In contrast, GRU(0.25) demonstrated significant differences in both CCC and NRMSE for all PK parameters between 5 × 10^4^ and 1 × 10^5^ and between 5 × 10^4^ and 2 × 10^5^; however, no significant differences were observed between 1 × 10^5^ and 2 × 10^5^ (Figure 3b,d).

#### 3.1.3 Performance of recurrent neural networks across varying number of hidden units

**FIGURE 4:**
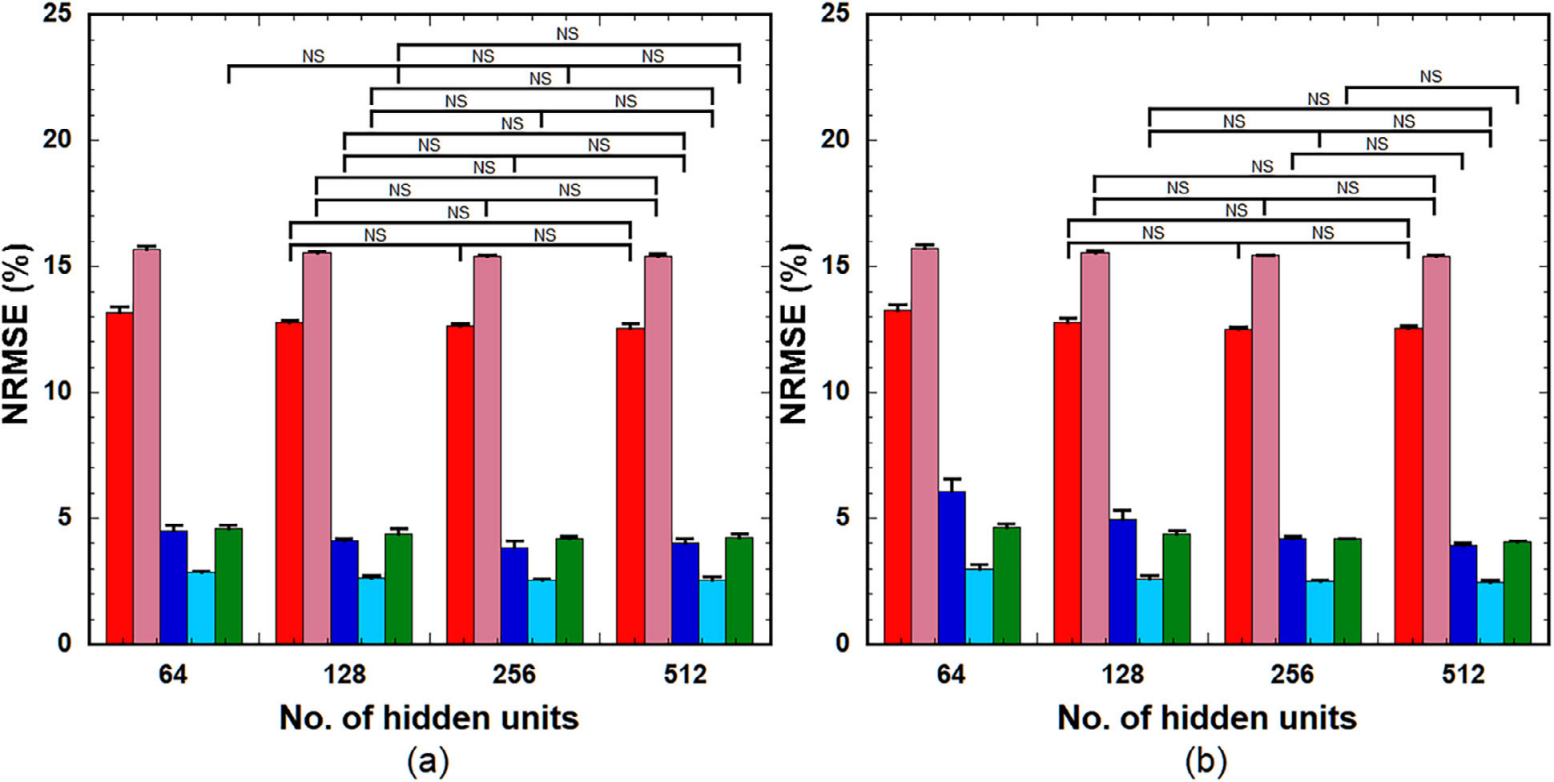
NRMSE of the PK parameters predicted using (a) LSTM(0.25) and (b) GRU(0.25) across varying numbers of hidden units (64, 128, 256, and 512) (left to right group). Red bar: *k*_ep_, pink bar: *v*_e_, blue bar: *v*_p_, light blue bar: *K* ^trans^, and green bar: *τ*_BAT_. Bar and error bars represent the mean and SD for *n* = 5, respectively. Significant differences were observed among parameters, excluding those indicated by ”NS” for each PK parameter. GRU: gated recurrent units; LSTM, long short‐term memory; NRMSE, normalized root mean square error; PK, pharmacokinetic; NS, not significant.

Figure 4 illustrates the NRMSE of the PK parameters predicted using (a) LSTM(0.25) and (b) GRU(0.25) across a varying number of hidden units (64, 128, 256, and 512) (left to right group). When using LSTM(0.25), significant differences in the NRMSE were observed for *k*_ep_, *v*_e_, *v*_p_, and *K* ^trans^ between 64 and 128, between 64 and 256, and between 64 and 512, and for *τ*_BAT_ between 64 and 256 and between 64 and 512. No significant differences were observed in other combinations (Figure 4a). GRU(0.25) exhibited significant differences in the NRMSE for *k*_ep_, *v*_e_, and *K* ^trans^ between 64 and 128, between 64 and 256, and between 64 and 512, and for *v*_p_ and *τ*_BAT_ between 64 and 128, between 64 and 256, between 64 and 512, between 128 and 256, and between 128 and 512. No significant differences were observed in other combinations (Figure 4b).

#### 3.1.4 Performance of recurrent neural networks when varying dropout rate at different peak signal‐to‐noise ratios

Figure 5a shows the CCC of the PK parameters predicted using the LSTMs (upper row) and GRUs (lower row) at different PSNRs, and Figure 5b shows their NRMSE. The left, middle, and right columns show the values for PSNRs at 10, 15, and 20–30, respectively. The left, middle, and right groups in each column show the values for DRs of 0, 0.25, and 0.5, respectively. When applying the LSTMs at PSNR = 10, the CCC values for LSTM(0.25) and LSTM(0.5) were significantly higher than those for LSTM(0) in *v*_p_, *K* ^trans^, and *τ*_BAT_ (Figure 5a, upper left). The NRMSE values for LSTM(0.25) and LSTM(0.5) were significantly lower than those for LSTM(0) in *v*_p_, *K* ^trans^, and *τ*_BAT_ (Figure 5b, upper left). When using GRUs, the CCC values for GRU(0.25) and GRU(0.5) were significantly higher than those for GRU(0) for *k*_ep_ and *v*_p_, and those for GRU(0.25) were significantly higher than those for GRU(0) for *K* ^trans^ (Figure 5a, lower left). The NRMSE values for GRU(0.25) and GRU(0.5) were significantly lower than those for GRU(0) for *k*_ep_, *v*_p_, and *K* ^trans^ (Figure 5b, lower left). When the PSNR was 15, the CCC values for LSTM(0.25) and LSTM(0.5) were significantly higher than those for LSTM(0) in *v*_p_ (Figure 5a, upper middle), and their NRMSE values were significantly lower than those for LSTM(0) in *v*_p_ (Figure 5b, upper middle). The CCC and NRMSE values for GRU(0.25) were significantly better than those for GRU(0) in *v*_p_ (Figures 5a,b, lower middle). When the PSNR was 20–30, the CCC for LSTM(0.5) was significantly lower than that for LSTM(0) for *k*_ep_ and *v*_e_ (Figure 5a, upper right). The NRMSE for LSTM(0.25) was smaller than that for LSTM(0) in *v*_p_; however, the difference between them did not reach statistical significance (*p* = 0.074) (Figure 5b, upper right). The CCC for GRU(0.25) was significantly higher than those for GRU(0) and GRU(0.5) in *v*_p_ (Figure 5a, lower right), and the NRMSE for GRU(0.25) was significantly lower than those for GRU(0) and GRU(0.5) in *v*_p_ (Figure 5b, lower right).

FIGURE 5(a) CCC and (b) NRMSE of the PK parameters predicted using the LSTM (upper row) and GRU (lower row) with DRs of 0, 0.25, and 0.5 (left to right group). Red bar: *k*_ep_, pink bar: *v*_e_, blue bar: *v*_p_, light blue bar: *K* ^trans^, and green bar: *τ*_BAT_. Bar and error bars represent the mean and SD for *n* = 5, respectively. No significant differences were observed among parameters, excluding those indicated by “*p* < 0.05” for each PK parameter. CCC, concordance correlation coefficient; DRs, dropout rates; GRU: gated recurrent units; LSTM, long short‐term memory; NRMSE, normalized root mean square error; PK, pharmacokinetic.

**Figure:**
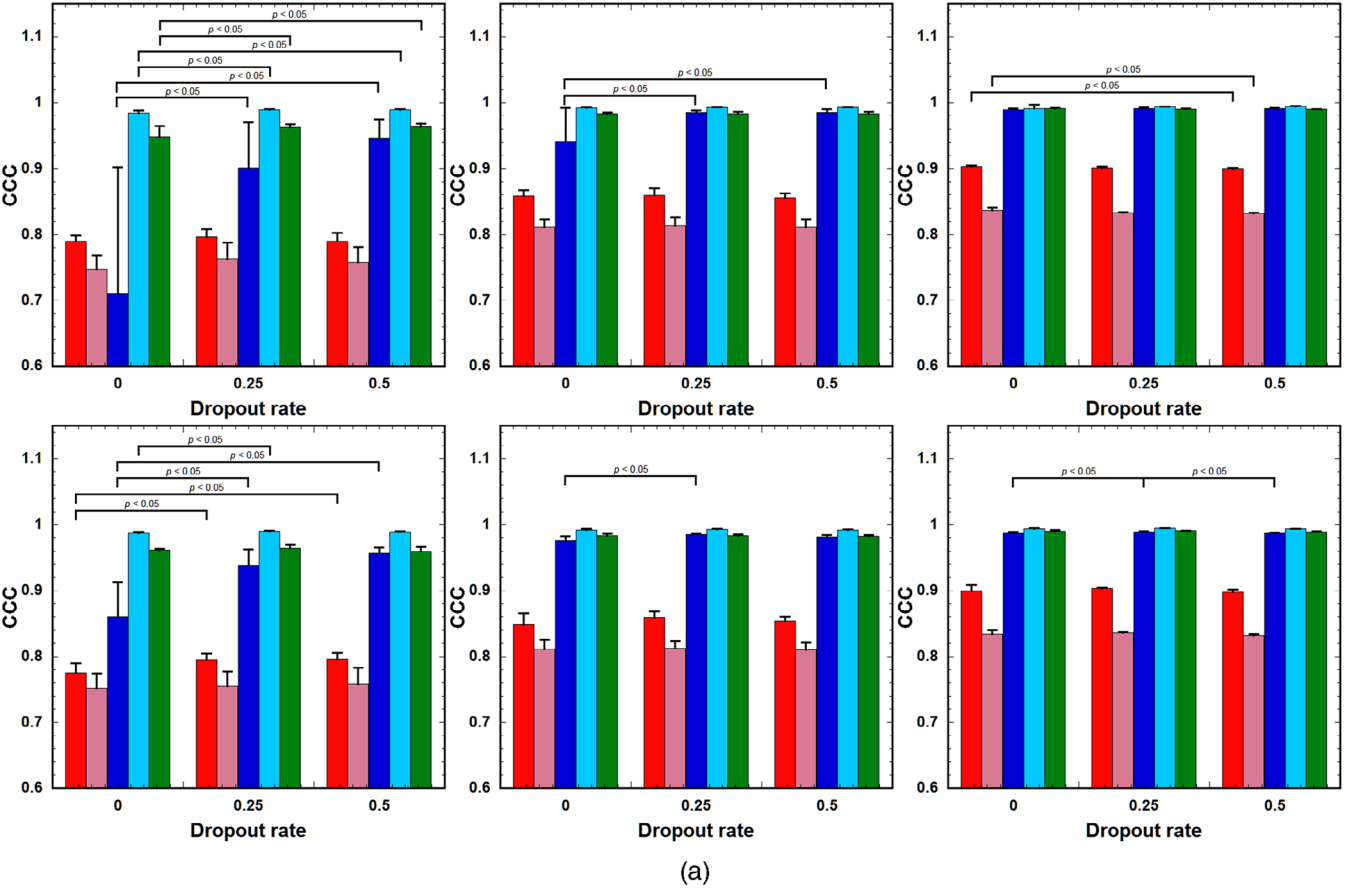

**Figure:**
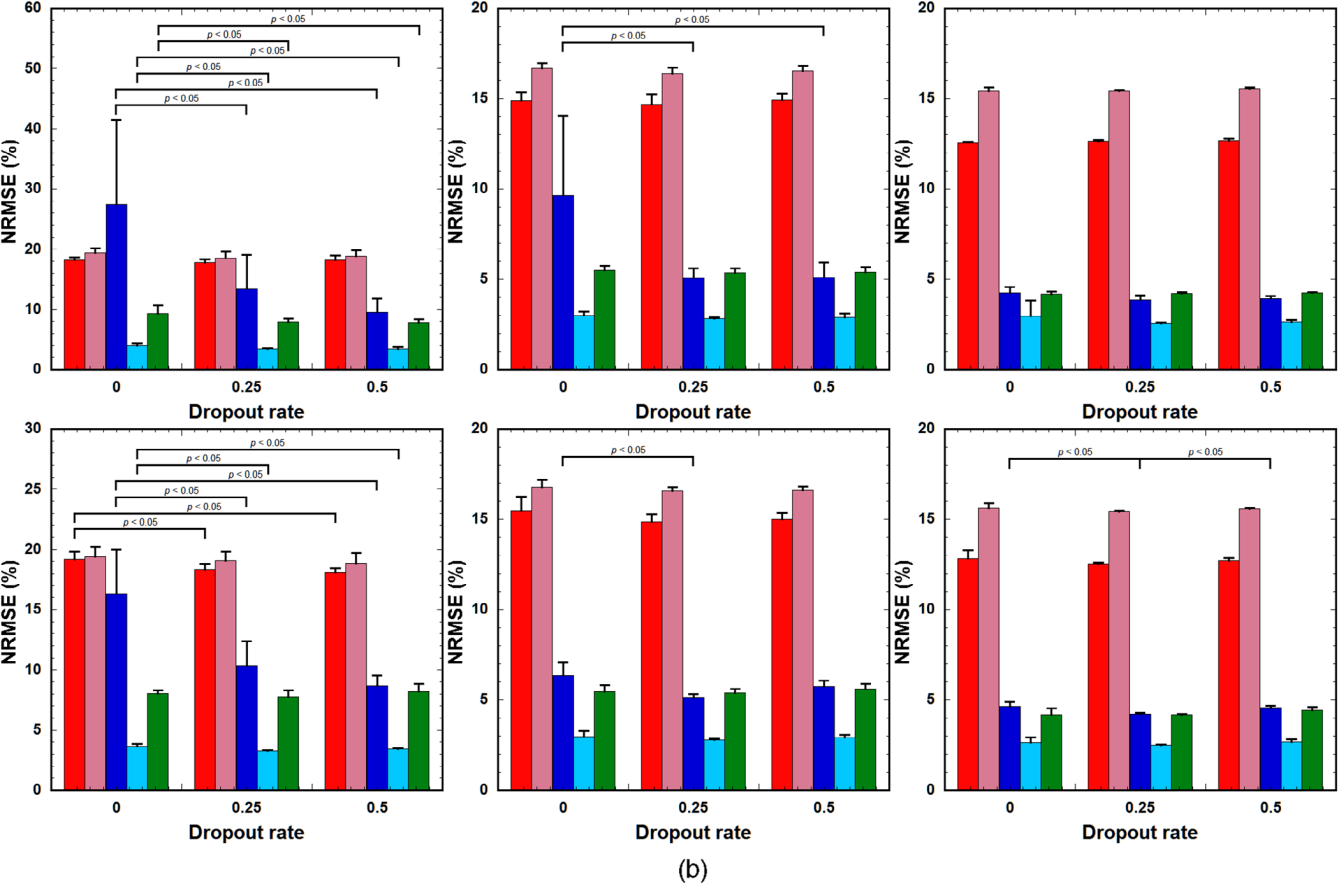

#### 3.1.5 Relationship between predictive uncertainty and dropout rate

**FIGURE 6:**
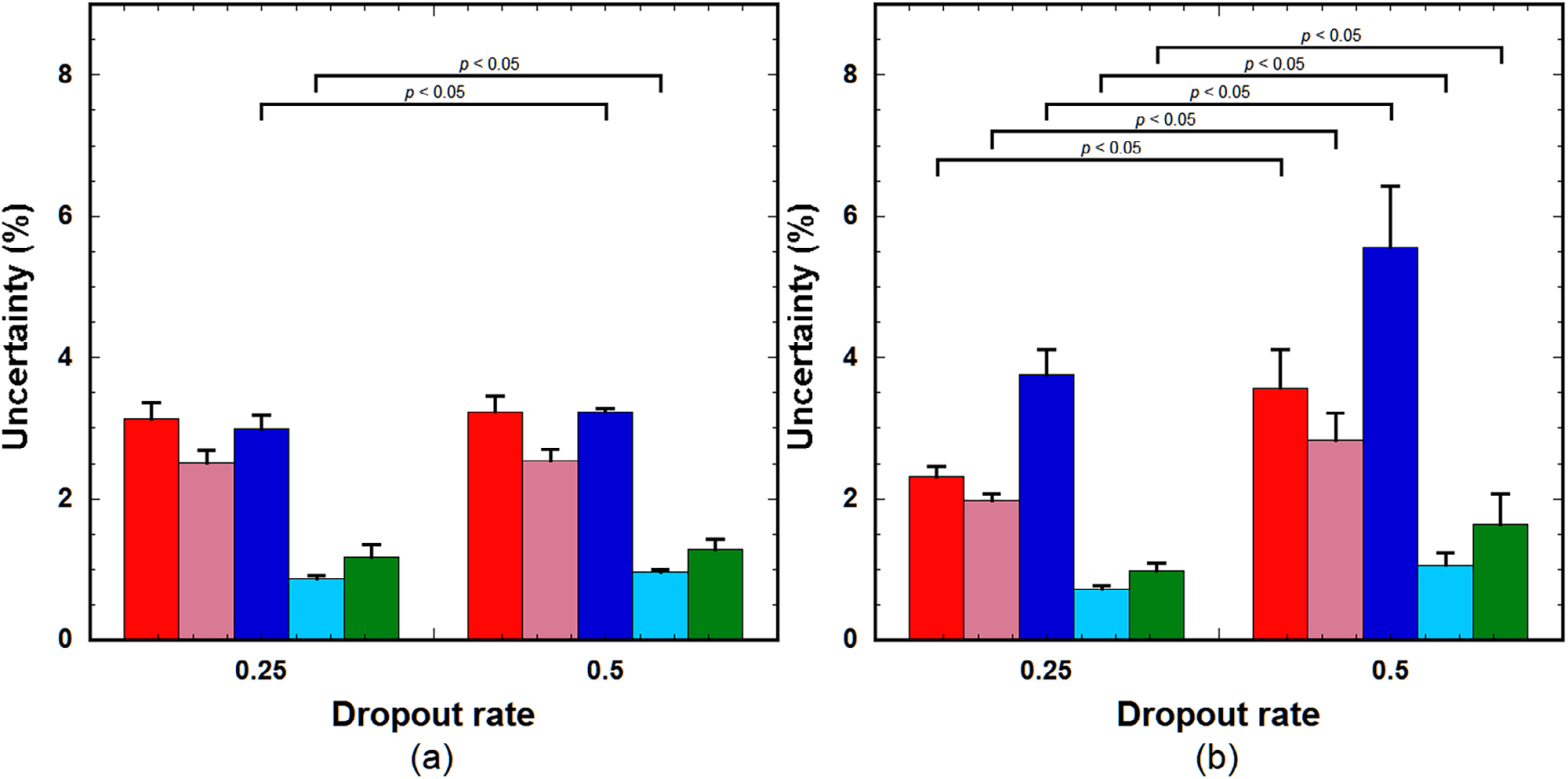
Uncertainties (Equation 4) of the PK parameters predicted using (a) LSTM and (b) GRU with DRs of 0.25 and 0.5. Red bar: *k*_ep_, pink bar: *v*_e_, blue bar: *v*_p_, light blue bar: *K* ^trans^, and green bar: *τ*_BAT_. Bar and error bars represent the mean and SD for *n* = 5, respectively. No significant differences were observed among parameters, excluding those indicated by “*p* < 0.05” for each PK parameter. DRs, dropout rates; GRU: gated recurrent units; LSTM, long short‐term memory; PK, pharmacokinetic.

Figure 6 shows the predictive uncertainties (Equation 4) of the PK parameters predicted using the (a) LSTM and (b) GRU with DRs of 0.25 and 0.5. The uncertainties in *v*_p_ and *K* ^trans^ predicted using LSTM(0.5) were significantly greater than those predicted using LSTM(0.25); however, there were no significant differences in the other PK parameters (Figure 6a). The uncertainties of all PK parameters predicted using GRU(0.5) were significantly greater than those predicted using GRU(0.25) (Figure 6b).

#### 3.1.6 Effect of bolus arrival time delay and dispersion in arterial input function

**FIGURE 7:**
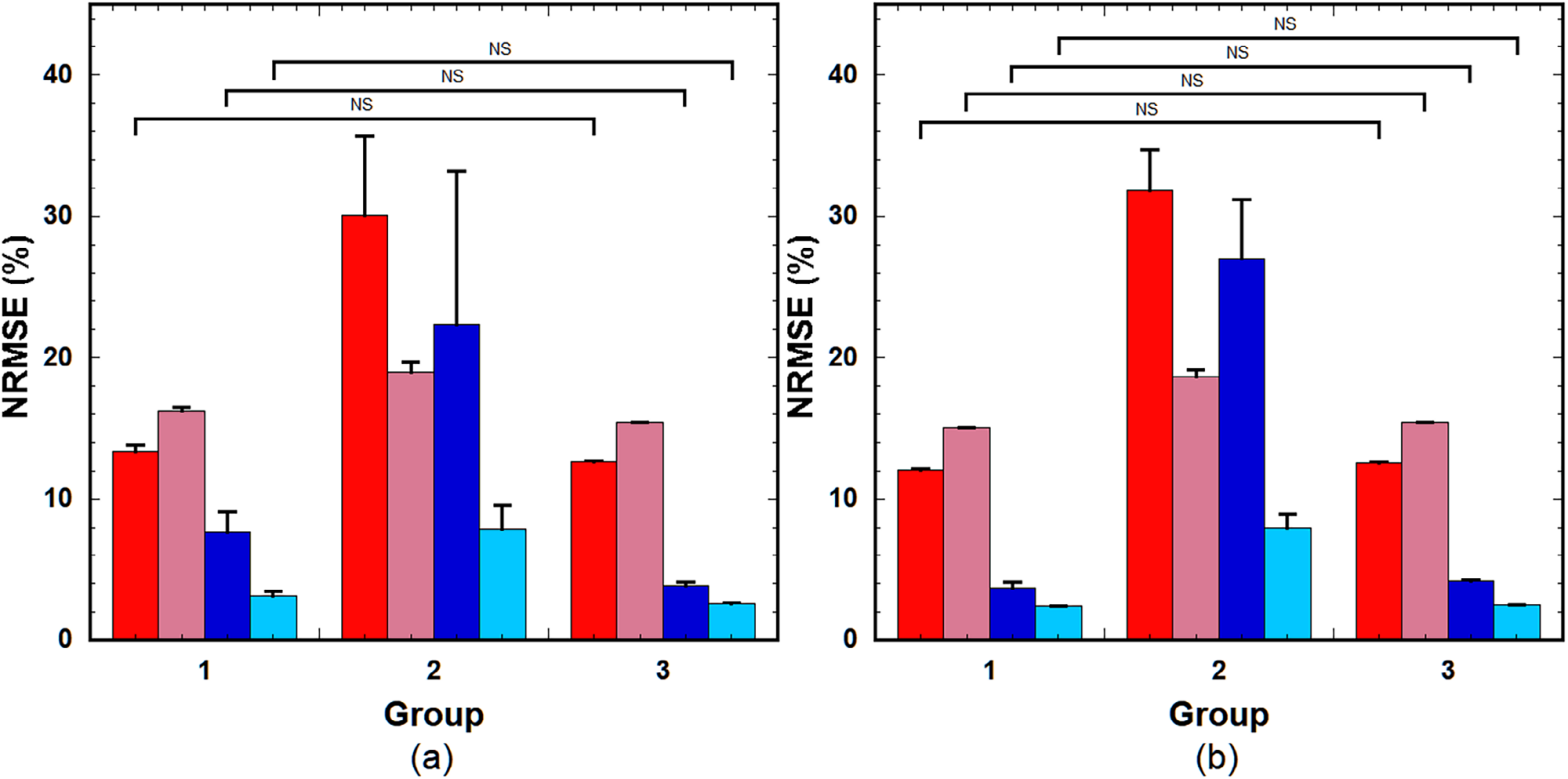
NRMSE of the PK parameters predicted using (a) LSTM(0.25) and (b) GRU(0.25). In Group 1, the RNNs trained using the samples without BAT delay in AIF were applied to the test samples without BAT delay. In Group 2, the RNNs trained using the samples without BAT delay in AIF were applied to the test samples with BAT delay. In Group 3, the RNNs trained using the samples with BAT delay in AIF were applied to the test samples with BAT delay. Red bar: *k*_ep_, pink bar: *v*_e_, blue bar: *v*_p_, and light blue bar: *K* ^trans^. Bar and error bars represent the mean and SD for *n* = 5, respectively. Significant differences were observed among parameters, excluding those indicated by ”NS” for each PK parameter. AIF, arterial input function; BAT, bolus arrival time; GRU: gated recurrent units; LSTM, long short‐term memory; NRMSE, normalized root mean square error; NS, not significant; PK, pharmacokinetic; RNN, recurrent neural network.

Figure 7 shows a comparison of the NRMSE among the groups with and without considering the BAT delay of the AIF in training and testing the RNNs ((a) for LSTM(0.25) and (b) for GRU(0.25)). In Group 1, the RNNs trained using samples without the BAT delay were applied to the test samples without the BAT delay. In Group 2, the RNNs trained using samples without the BAT delay were applied to the test samples with the BAT delay. In Group 3, the RNNs trained using samples with the BAT delay were applied to the test samples with the BAT delay. As shown in Figure 7a, when using LSTM(0.25), the NRMSE in Group 2 was significantly higher than that in Group 1 for all PK parameters. In contrast, the NRMSE values for *k*_ep_, *v*_p_, and *K* ^trans^ in Group 3 decreased to a level that was not significantly different from those in Group 1, and the value for *v*_e_ was significantly smaller than that in Group 1. When using GRU(0.25) (Figure 7b), the NRMSE values in Group 2 were significantly larger than those in Group 1 for all PK parameters, and those in Group 3 decreased to a level with no significant difference from those in Group 1.

**FIGURE 8:**
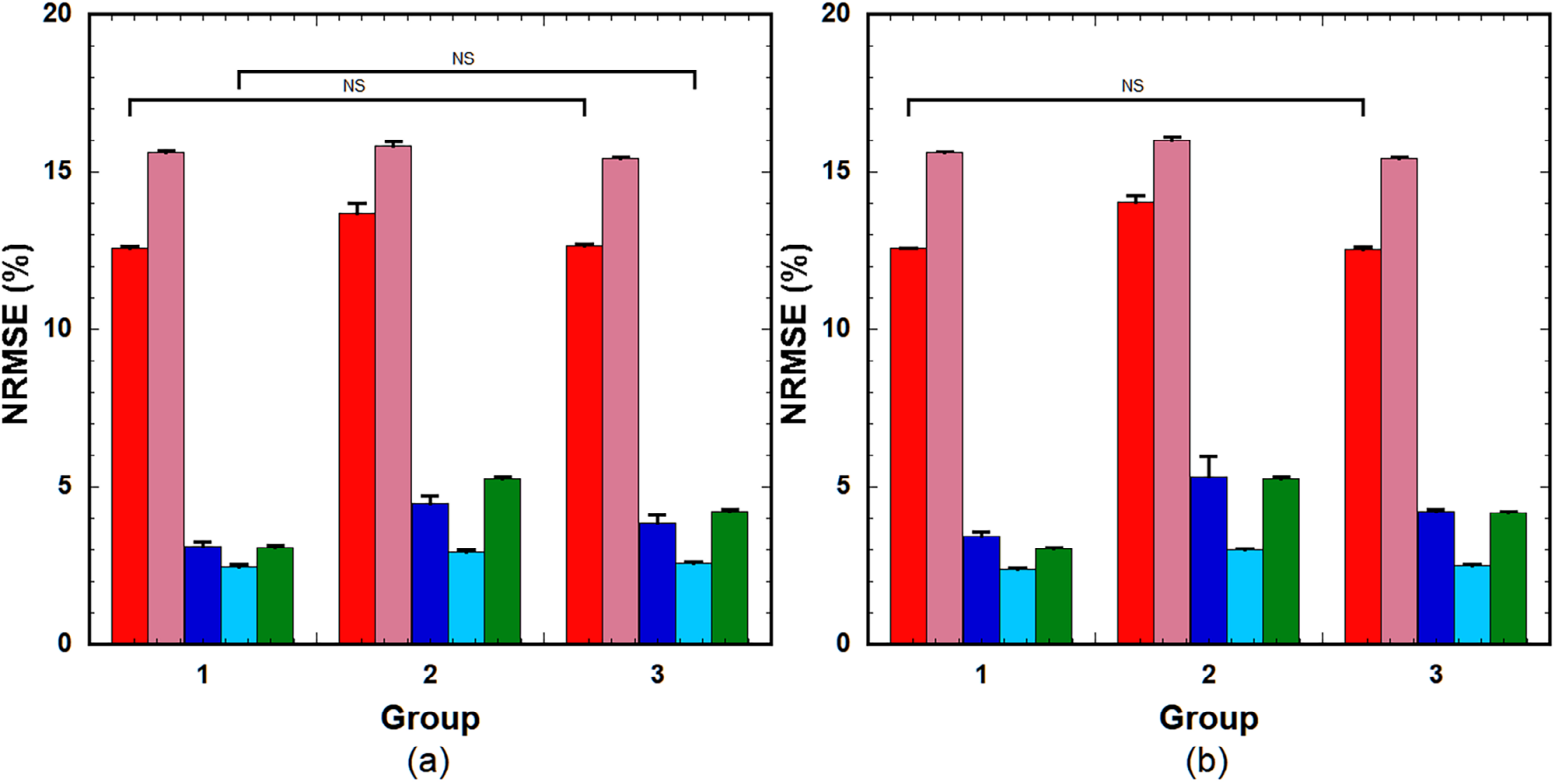
NRMSE of the PK parameters predicted using (a) LSTM(0.25) and (b) GRU(0.25). In Group 1, the RNNs trained using the samples without the dispersion in AIF were applied to the test samples without the AIF dispersion. In Group 2, the RNNs trained using the samples without the dispersion were applied to the test samples with the dispersion. In Group 3, the RNNs trained using the samples with the AIF dispersion were applied to the test samples with the dispersion. Red bar: *k*_ep_, pink bar: *v*_e_, blue bar: *v*_p_, light blue bar: *K* ^trans^, and green bar: *τ*_BAT_. Bar and error bar represent the mean and SD for *n* = 5, respectively. Significant differences were observed among parameters, excluding those indicated by ”NS” for each PK parameter. AIF, arterial input function; GRU: gated recurrent units; LSTM, long short‐term memory; NRMSE, normalized root mean square error; NS, not significant; PK, pharmacokinetic; RNN, recurrent neural network.

Figure 8 shows a comparison of the NRMSE among the groups with and without considering the AIF dispersion in training and testing the RNNs ((a) for LSTM(0.25) and (b) for GRU(0.25)). In Group 1, the RNNs trained using samples without AIF dispersion were applied to the test samples without dispersion. In Group 2, the RNNs trained using samples without dispersion were applied to the test samples with dispersion. In Group 3, the RNNs trained using samples with AIF dispersion were applied to the test samples with AIF dispersion. As shown in Figure 8a, the NRMSE values in Group 2 were significantly higher than those in Group 1 for all the PK parameters. In contrast, no significant differences in *k*_ep_ and *K* ^trans^ were observed between Groups 1 and 3. The NRMSE for *v*_e_ in Group 3 was significantly smaller than that in Group 1, and those for *v*_p_ and *τ*_BAT_ were significantly larger than those in Group 1. When using GRU(0.25) (Figure 8b), the NRMSE values in Group 2 were significantly larger than those in Group 1 for all PK parameters. There was no significant difference in *k*_ep_ between groups 1 and 3. The NRMSE for *v*_e_ in Group 3 was significantly smaller than that in Group 1, and those for *v*_p_, *K* ^trans^, and *τ*_BAT_ in Group 3 were significantly larger than those in Group 1.

#### 3.1.7 Comparison of long short‐term memory, gated recurrent units, and nonlinear least‐squares method performances

**FIGURE 9:**
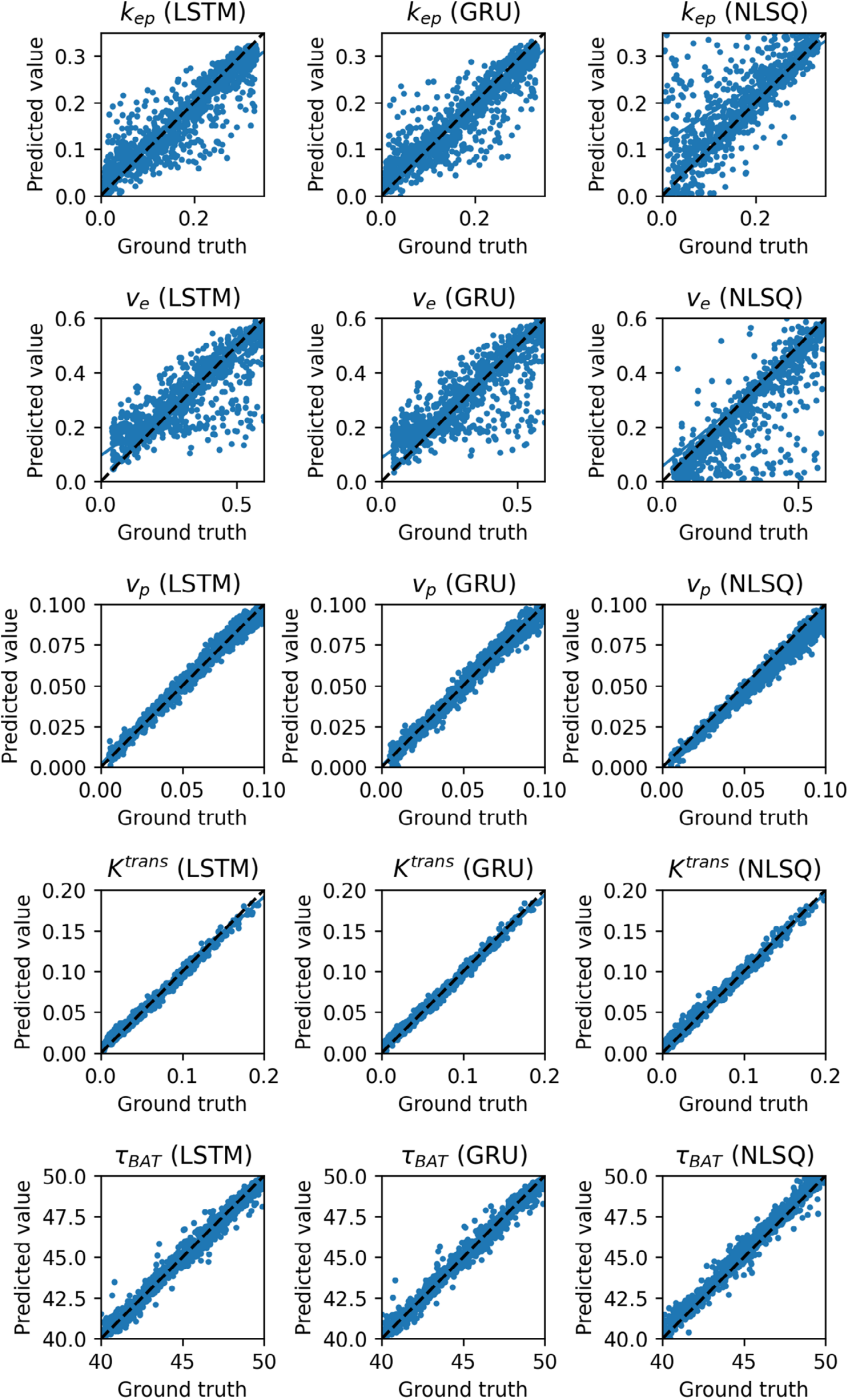
Correlations between the predicted values of the PK parameters (*k*_ep_, *v*_e_, *v*_p_, *K* ^trans^, and *τ*_BAT_ from top to bottom row) and corresponding ground truth values for 1000 samples. The left, middle, and right columns show the values for LSTM(0.25), GRU(0.25), and NLSQ methods, respectively. The dashed line represents the line of identity. GRU: gated recurrent units; LSTM, long short‐term memory; NLSQ, nonlinear least‐squares; PK, pharmacokinetic.

Figure 9 shows the correlations between the predicted and ground truth values for *k*_ep_, *v*_e_, *v*_p_, *K* ^trans^, and *τ*_BAT_ (top to bottom row). The left, middle, and right columns show the cases using LSTM(0.25), GRU(0.25), and NLSQ, respectively. As shown in Figure 9, the scatter and bias of the *k*_ep_ and *v*_e_ values obtained by NLSQ are larger than those obtained by LSTM(0.25) and GRU(0.25). Compared to the ground truth, the *k*_ep_ values obtained by NLSQ were biased towards larger values, whereas the *v*_e_ values were biased in the opposite direction. The scatter and bias for *K* ^trans^ were the smallest among the PK parameters for all the methods.

**FIGURE 10:**
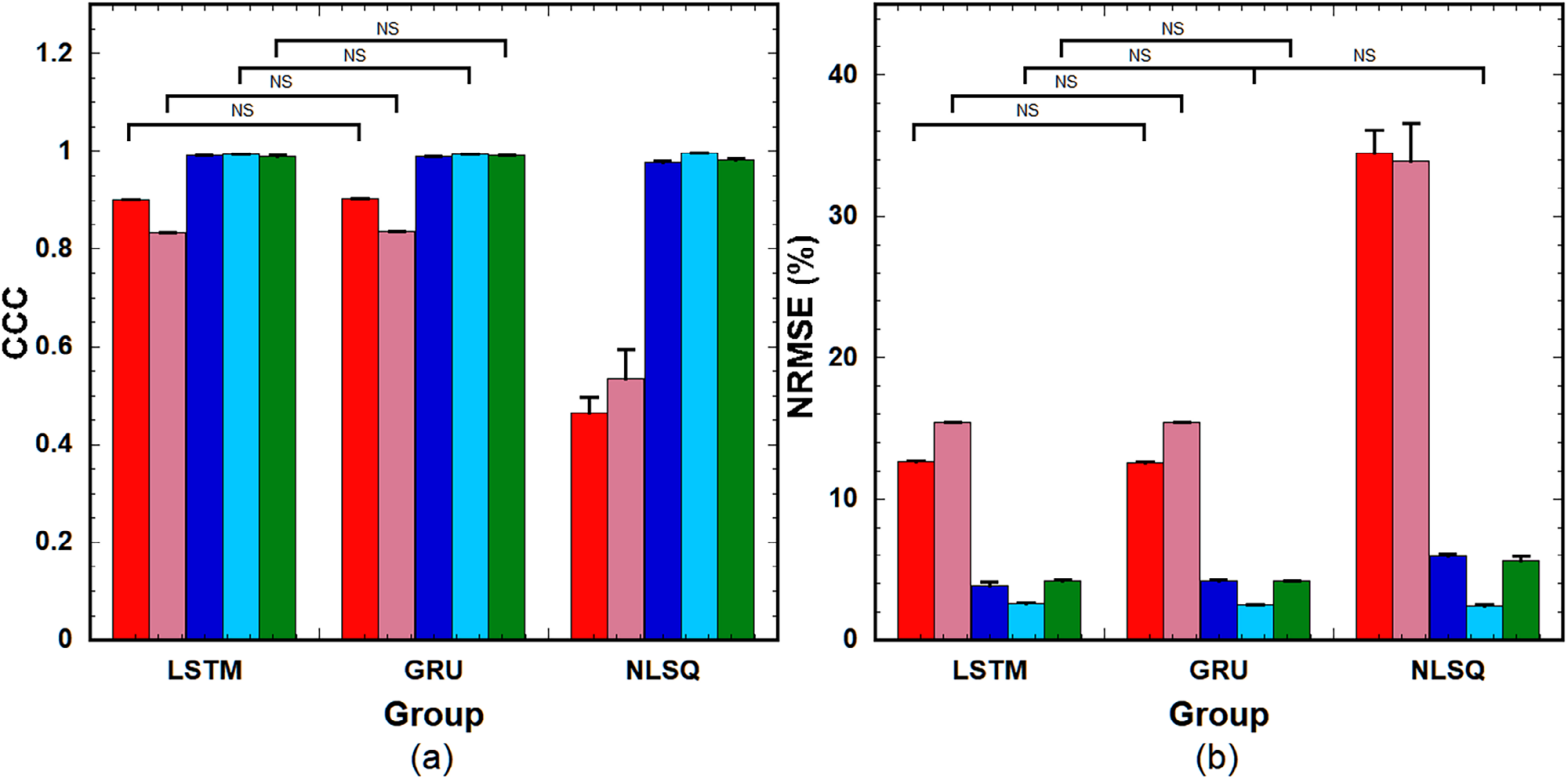
(a) CCC and (b) NRMSE of the PK parameters predicted using LSTM(0.25), GRU(0.25), and NLSQ (left to right group). Red bar: *k*_ep_, pink bar: *v*_e_, blue bar: *v*_p_, light blue bar: *K* ^trans^, and green bar: *τ*_BAT_. Bar and error bars represent the mean and SD for *n* = 5, respectively. Significant differences were observed among parameters, excluding those indicated by “NS” for each PK parameter. CCC, concordance correlation coefficient; GRU: gated recurrent units; LSTM, long short‐term memory; NRMSE, normalized root mean square error; NLSQ, nonlinear least‐squares; NS, not significant; PK, pharmacokinetic.

Figure 10a shows the CCC of the PK parameters obtained using LSTM(0.25), GRU(0.25), and NLSQ (left to right group), and Figure 10b shows their corresponding NRMSE. As shown in Figure 10a, there were no significant differences between LSTM(0.25) and GRU(0.25) in the PK parameters, except for *v*_p_, and the CCC of *v*_p_ for LSTM(0.25) was significantly higher than that for GRU(0.25). In contrast, when using NLSQ, the CCC values for the PK parameters, except for *K* ^trans^ were significantly lower than those for LSTM(0.25) and GRU(0.25), whereas those for *K* ^trans^ were significantly higher than those for LSTM(0.25) and GRU(0.25). Regarding the NRMSE, no significant differences in PK parameters were observed between LSTM(0.25) and GRU(0.25), except for *v*_p_, and the NRMSE of *v*_p_ for LSTM(0.25) was significantly lower than that for GRU(0.25) (Figure 10b). When using NLSQ, the NRMSE values for the PK parameters, except for *K* ^trans^ were significantly larger than those for LSTM(0.25) and GRU(0.25), whereas the NRMSE for *K* ^trans^ was significantly smaller than that for LSTM(0.25) and did not significantly differ from that for GRU(0.25).

#### 3.1.8 Comparison of computation time

When 10^5^ training samples and 256 hidden units were used, the computation time for training LSTM(0.25) was 5648 ± 165 s (mean ± SD for *n* = 5), which was significantly higher than that for GRU(0.25) (4883 ± 71 s).

The computation times for processing 1000 test samples were as follows: Trained LSTM without MCD, (LSTM(0)) and GRU(0) required 0.67 ± 0.17 s and 0.71 ± 0.16 s, respectively, with no significant difference between them. When applying the trained LSTM(0.25) to 1000 test samples and running it 100 times for each test sample to quantify the uncertainty, the computation time was 142 ± 16 s, which did not significantly differ from those for the trained LSTM(0.5) (141 ± 16 s), GRU(0.25) (128 ± 20 s), and GRU(0.5) (124 ± 16 s), and there was no significant difference between the GRU(0.25) and GRU(0.5). The computation times for these methods were significantly less than those for NLSQ (168 ± 12 s).

### 3.2 Clinical study

#### 3.2.1 Pharmacokinetic parameter and predictive uncertainty images

Figure 11a shows examples of PK parameters (first, second, and fourth columns) and predictive uncertainty images (third and fifth columns) generated using the LSTMs. For comparison, the PK parameter images obtained using NLSQ are shown in the right column. Figure 11b shows the results for the GRUs and NLSQ. In this example, the *v*_p_ and *τ*_BAT_ images obtained by the LSTMs varied significantly depending on the DR, whereas the *k*_ep_, v_e_, and *K* ^trans^ images showed no significant differences (Figure 11a). No significant differences were observed between the *k*_ep_ images obtained using LSTMs, which exhibited significantly higher quality than those using NLSQ. Both LSTM(0.25) and LSTM(0.5) produced superior *v*_e_ images with reduced noise than those obtained using LSTM(0) and NLSQ. The quality of the *v*_p_ image obtained using LSTM(0.25) was better than those obtained using LSTM(0) and LSTM(0.5) and was comparable to NLSQ. No significant differences were observed in *K* ^trans^ images between the methods. The *τ*_BAT_ images obtained using LSTM(0.25) and LSTM(0.5) were similar and exhibited reduced noise than those obtained using NLSQ. The pixel values of the *τ*_BAT_ image for LSTM(0) were higher than those for LSTM(0.25), LSTM(0.5), and NLSQ. When using the GRUs (Figure 11b), the PK parameter images, except for *K* ^trans^ varied significantly depending on the DR. The *k*_ep_ image obtained using GRU(0.5) exhibited less noise than that obtained using GRU(0) and slightly better than that obtained using GRU(0.25). Overall, GRU models generated *k*_ep_ images with much less noise than those generated using NLSQ. The *v*_e_ images obtained using GRU(0.25) and GRU(0.5) were similar and slightly less noisy than those obtained using GRU(0). The *v*_e_ images obtained by the GRUs were much less noisy than those obtained using NLSQ. Although all *v*_p_ images obtained using the GRUs were slightly inferior to those obtained using NLSQ, GRU(0.5) generated the best images among all the GRUs. Similar to the LSTMs, there were no significant differences in the *K* ^trans^ images between the methods. The *τ*_BAT_ images obtained using GRU(0.25) and GRU(0.5) were similar and exhibited reduced noise than those obtained using NLSQ. The pixel values of the *τ*_BAT_ image for GRU(0) were considerably lower than those for GRU(0.25), GRU(0.5), and NLSQ.

FIGURE 11(a) PK parameter (*k*_ep_, *v*_e_, *v*_p_, *K* ^trans^, and *τ*_BAT_ from top to bottom row) and predictive uncertainty (*σ*_uc_) images generated using the LSTM with different DRs. The first, second, and fourth columns show the PK parameter images obtained using LSTM(0), LSTM(0.25), and LSTM(0.5), respectively. The third and fifth columns show the *σ*_uc_ images obtained using LSTM(0.25) and LSTM(0.5), respectively. For comparison, the PK parameter images obtained by the NLSQ are shown in the right column. (b) PK parameter (*k*_ep_, *v*_e_, *v*_p_, *K* ^trans^, and *τ*_BAT_ from top to bottom row) and predictive uncertainty (*σ*_uc_) images generated using the GRU with different DRs. The first, second, and fourth columns show the PK parameter images obtained using GRU(0), GRU(0.25), and GRU(0.5), respectively. The third and fifth columns show the *σ*_uc_ images obtained using GRU(0.25) and GRU(0.5), respectively. For comparison, the PK parameter images obtained by the NLSQ are shown in the right column. DRs, dropout rates; GRU: gated recurrent units; LSTM, long short‐term memory; NLSQ, nonlinear least‐squares; PK, pharmacokinetic.

**Figure:**
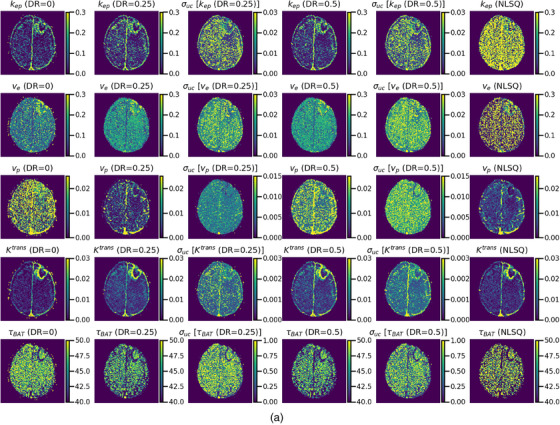

**Figure:**
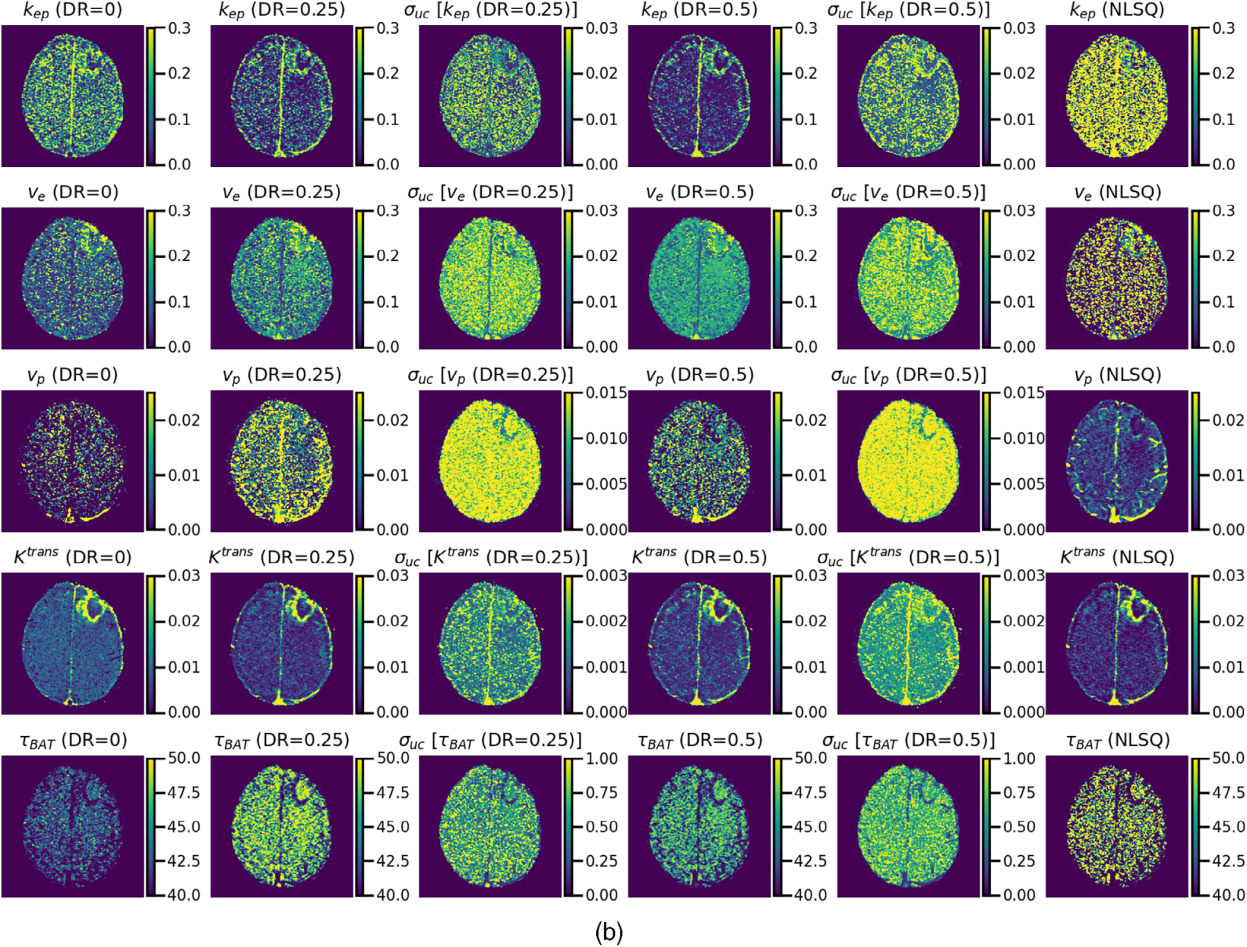

When comparing the predictive uncertainty images obtained by the LSTMs (third and fifth columns in Figure 11a) and GRUs (third and fifth columns in Figure 11b), the predictive uncertainties obtained using the GRUs were generally greater than those obtained using the LSTMs. The uncertainty images of the PK parameters, except for *v*_p_ were similar to the corresponding PK parameter images for both LSTMs and GRUs; however, the pixel value variations in the uncertainty images were not as large as those in the PK parameter images.

#### 3.2.2 Comparison of concordance correlation coefficients for pharmacokinetic parameters in tumors

**FIGURE 12:**
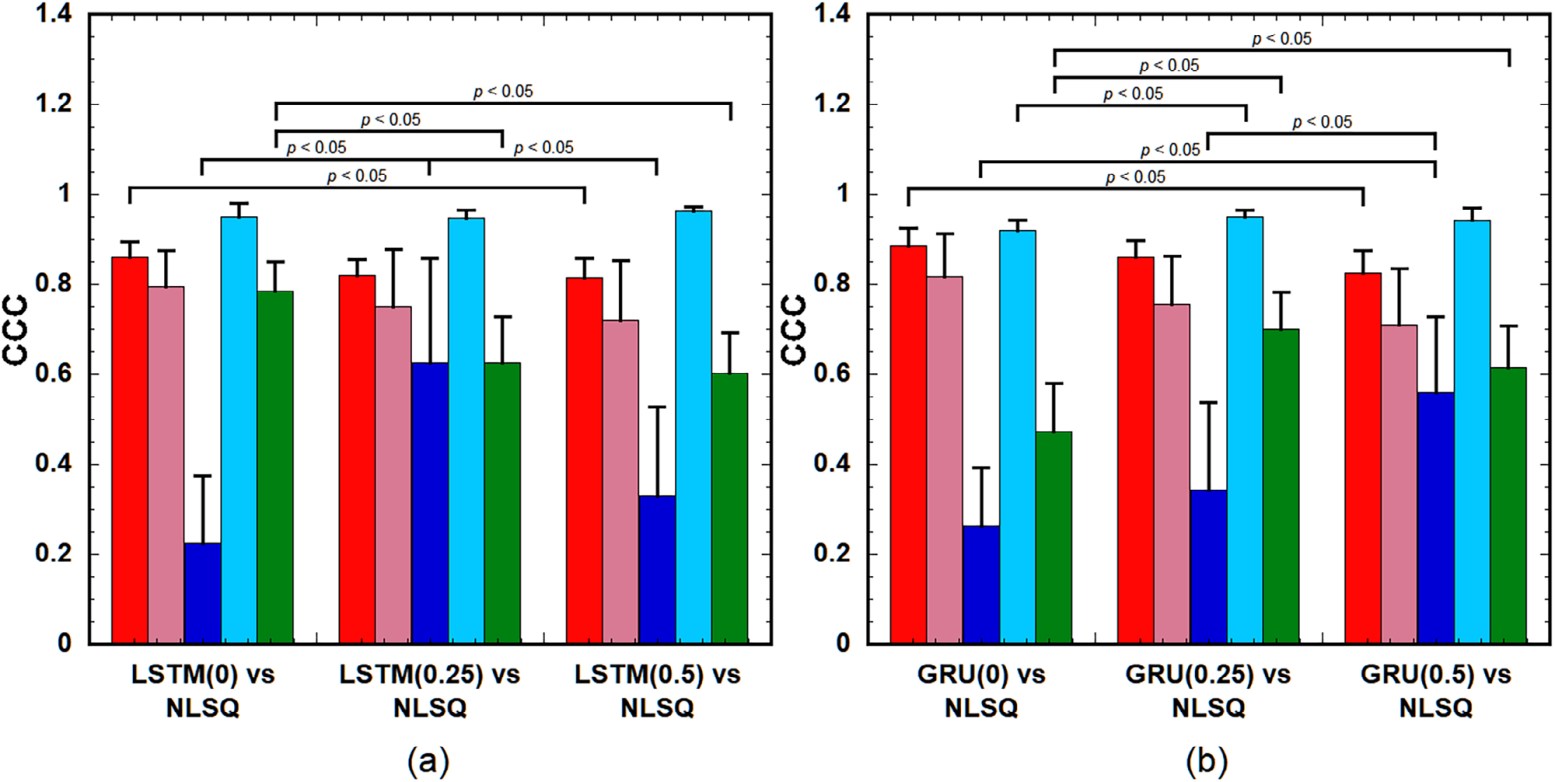
(a) CCC of the PK parameters in tumors for various combinations of the LSTM with different DRs and NLSQ. (b) CCC of the PK parameters in tumors for various combinations of the GRU with different DRs and NLSQ. Red bar: *k*_ep_, pink bar: *v*_e_, blue bar: *v*_p_, light blue bar: *K* ^trans^, and green bar: *τ*_BAT_. Bar and error bars represent the mean and SD for *n* = 8, respectively. No significant differences were observed among parameters, excluding those indicated by “*p* < 0.05” for each PK parameter. CCC, concordance correlation coefficient; DRs, dropout rates; GRU: gated recurrent units; LSTM, long short‐term memory; NLSQ, nonlinear least‐squares; PK, pharmacokinetic.

Figure 12a,b show the CCC values of the PK parameters in the tumors for various combinations of LSTMs and NLSQ and combinations of GRUs and NLSQ, respectively. As shown in Figure 12a, the CCC of *k*_ep_ between LSTM(0) and NLSQ was significantly higher than that between LSTM(0.5) and NLSQ. The CCC of *v*_p_ between LSTM(0.25) and NLSQ was significantly higher than that between LSTM(0) and NLSQ and between LSTM(0.5) and NLSQ. The CCC of *τ*_BAT_ between LSTM(0) and NLSQ was significantly higher than that between LSTM(0.25) and NLSQ and between LSTM(0.5) and NLSQ. No significant differences were observed among the other combinations. When using GRUs, the CCC of *k*_ep_ between GRU(0) and NLSQ was significantly higher than that between GRU(0.5) and NLSQ (Figure 12b). The CCC of *v*_p_ between the GRU(0.5) and NLSQ was significantly higher than that between GRU(0) and NLSQ and between GRU(0.25) and NLSQ. The CCC of *K* ^trans^ between GRU(0.25) and NLSQ was significantly higher than that between GRU(0) and NLSQ. The CCC values of *τ*_BAT_ between GRU(0.25) and NLSQ and between GRU(0.5) and NLSQ were significantly higher than those between GRU(0) and NLSQ. No significant differences were observed among the other combinations.

#### 3.2.3 Comparison of predictive uncertainties for pharmacokinetic parameters in tumors

Table 1 summarizes the predictive uncertainties of PK parameters in tumors when using LSTMs and GRUs. As shown in Table 1, the predictive uncertainty for the LSTM did not change significantly depending on the DR, whereas that for the GRU increased significantly with increasing DR (indicated by *). The predictive uncertainty for GRU(0.5) was significantly greater than that for LSTM(0.5) in the PK parameters except for *τ*_BAT_ (indicated by **).

**TABLE 1:** Summary of the predictive uncertainties of the pharmacokinetic parameters in tumors using LSTM and GRU with different DRs.

RNNs(DR)	*k*_ep_ (min^−1^)	*v*_e_	*v*_p_	*K* ^trans^ (min^−1^)	*τ*_BAT_ (s)
LSTM(0.25)	0.0101 ± 0.0027	0.0140 ± 0.0049	0.00550 ± 0.00330	0.00144 ± 0.00035	0.307 ± 0.090
LSTM(0.5)	0.0107 ± 0.0026^**^	0.0146 ± 0.0038^**^	0.00617 ± 0.00163^**^	0.00164 ± 0.00031^**^	0.269 ± 0.078
GRU(0.25)	0.0094 ± 0.0017^*^	0.0154 ± 0.0042^*^	0.00849 ± 0.00226^*^	0.00161 ± 0.00026^*^	0.244 ± 0.053^*^
GRU(0.5)	0.0139 ± 0.0027^*^, ^**^	0.0196 ± 0.0045^*^, ^**^	0.00877 ± 0.00160^*^, ^**^	0.00242 ± 0.00054^*^, ^**^	0.315 ± 0.059^*^

*Note*: Data are presented as the mean ± SD for *n* = 8. LSTM(0.25) and LSTM(0.5) denote LSTM with DRs of 0.25 and 0.5, respectively, and GRU(0.25) and GRU(0.5) denote GRU with DRs of 0.25 and 0.5, respectively.

Abbreviations: DR, dropout rate; GRU: gated recurrent units; LSTM, long short‐term memory; RNNs, recurrent neural networks.

* *p* < 0.05, between GRU(0.25) and GRU(0.5)

** *p* < 0.05, between LSTM(0.5) and GRU(0.5)

## 4 DISCUSSION

This study quantitatively evaluated the performance of two types of RNNs (LSTM and GRU) incorporated with MCD for predicting PK parameters from DCE‐MRI data through simulation and clinical studies. The findings of this study may improve our understanding of RNN performance in terms of PK parameter prediction accuracy and reliability. Additionally, the findings will guide the selection of hyperparameters of the RNN architecture and MCD that are suitable for PK parameter prediction from DCE‐MRI data.

The GRU architecture is simpler than that of the LSTM, with one gate less than the LSTM, which reduces matrix multiplication, thereby resulting in a lower computational cost than the LSTM. The computation time for training the GRU was significantly less than that for the LSTM, as described in the “Results” section. Our findings indicate that LSTM outperforms GRU when the number of training samples is small (Figure 3). Furthermore, compared to LSTM, the prediction accuracy of the GRU depends more on the number of hidden units (Figure 4). To achieve comparable prediction accuracy, GRU requires more hidden units than LSTM. Cahuantzi et al.^28^ reported that GRU outperformed LSTM on low‐complexity sequences in natural language processing, whereas LSTM performed better on high‐complexity sequences owing to their ability to capture long‐term dependencies. They also reported the number of units per layer as a critical hyperparameter.^28^ Although their study objective is different from ours, the similarities between the results could be attributed to the structural differences between LSTM and GRU, particularly the number of gates between LSTM and GRU.

As shown in Figure 2, without the MCD, large differences between the training and validation losses were observed when the number of epochs was less than approximately 40 in both the LSTM and GRU. These differences could be attributed to overfitting. In contrast, when using the MCD, no such differences were observed even at small epoch numbers in both the LSTM and GRU, suggesting that the MCD can help suppress overfitting and improve training stability. Our simulation results (Figure 5) further support the role of MCD in enhancing or preserving the prediction accuracy of the PK parameters when the DR is adjusted according to the noise level of the DCE‐MRI data. Specifically, a DR of 0.25–0.5 is recommended at low PSNR, and 0.25 or smaller is suitable for other PSNRs. Moreover, our findings (Figure 11) suggest that MCD improves the quality of PK parameter images. However, in clinical studies, it is difficult to draw definitive conclusions because the ground truth is not known, unlike in simulation studies, and the results are significantly affected by tumor size, type, and malignancy. When comparing the PK parameter images (Figure 11) and the CCC values for LSTMs, GRUs, and NLSQ (Figure 12), the recommended DR for LSTM and GRU is 0.25 and 0.5, respectively.

MCD is also useful for quantifying the uncertainty of predicted PK parameters; however, the computation time was significantly higher, although not higher than the NLSQ method. When using the LSTM, the predictive uncertainty did not significantly change depending on the DR but increased with increasing DR when using the GRU (Figure 6). These findings align with our clinical study findings (Table 1), which could be attributed to the difference in the network architecture between LSTM and GRU.

Verdoja et al.^29^ theoretically investigated the behavior of MCD using a simple single‐layer network model and established a proportional relationship between the variance of the posterior probability distribution and the square of the average ground truth value; that is, the larger the value to be predicted, the greater the uncertainty. Our study findings corroborated their findings in many cases, as the uncertainty images were similar to the predicted PK parameter images (Figure 11). However, there were some discrepancies, particularly in the *v*_p_ images, warranting further investigation using more complex network models to elucidate the underlying factors.

As shown in Figures 7 and 8, although the effect of BAT delay on AIF was greater than that of dispersion, it could be effectively mitigated by incorporating BAT delay into RNN training. Although the effect of AIF dispersion was not as substantial as that of the BAT delay effect, it could not be sufficiently corrected, even when incorporated into RNN. This difficulty also applies to the quantification of cerebral blood flow using dynamic susceptibility contrast MRI.^21^, ^30^ To address the AIF dispersion effect, it may be necessary to use a tracer kinetic model that explicitly includes this effect and to add $\tau_{d}$ to the output parameters in RNNs, which will be the focus of our future research.

Our findings revealed no significant differences between the *K* ^trans^ images obtained using the RNNs and NLSQ (Figure 11). In addition, unlike the *v*_p_ images, the quality of the *K* ^trans^ images obtained by LSTM and GRU did not significantly depend on the DR. These findings align with the simulation results (Figure 5). In contrast to tumor regions, brain regions with an intact blood‐brain barrier exhibited significant differences in *k*_ep_ and *v*_e_ images between RNNs with MCD and NLSQ (Figure 11). Moreover, visual inspection of NLSQ‐generated *k*_ep_ and *v*_e_ images revealed an apparent interdependence rather than independence, aligning with the observed opposite biases in the correlation between the estimated and ground truth values of these parameters (Figure 9). From a physiological perspective, it is difficult to explain the behavior of these parameters. Furthermore, the evaluation metrics (CCC and NRMSE) of the PK parameters, except for *K* ^trans^ obtained using NLSQ, were significantly inferior to those obtained using the RNNs with MCD (Figure 10). In addition, the accuracy of the NLSQ method is affected by statistical noise and initial estimates,^6^ which may also be responsible for these discrepancies. Based on these findings, RNNs with MCD are considered to outperform NLSQ.

Moreover, our findings (Figure 11) indicated that the *v*_e_ images obtained using RNNs with MCD exhibited changes in parallel with the *k*_ep_ and *K* ^trans^ images of the tumor. In contrast, the *v*_p_ images showed different patterns, increasing around the tumor and decreasing inside the tumor. Although a comparison with pathological studies is necessary for an accurate evaluation, these increases and decreases appear to be due to angiogenesis and necrosis and/or underestimation due to the large extravascular extracellular space within the tumor, respectively. Therefore, *v*_p_​ could serve as a valuable biomarker for understanding the pathophysiological state of tumors and evaluating their therapeutic effects and outcomes.

In this study, *τ*_BAT_ was added as an output parameter in the RNNs. In many cases, *τ*_BAT_ exhibited a tendency to decrease in regions of increased *K* ^trans^ and to increase within the tumor (Figure 11). It appears that a decrease in *τ*_BAT_ reflects hyperpermeability and/or disruption of the blood‐brain barrier, and its increase reflects necrosis in tumors. Therefore, *τ*_BAT_ and *K* ^trans^ appear to be useful for evaluating the pathophysiological state of the blood‐brain barrier and tumors. However, reducing the number of trainable parameters may be effective for reducing the burden on RNNs, such as the number of necessary training samples. Investigating the optimal number of output parameters will be one of our future research subjects.

In this study, we used the population‐averaged AIF for both simulation and clinical investigations. These population‐averaged AIFs have often been used instead of individual AIFs in clinical settings.^31^, ^32^ Although individual AIFs are more patient‐specific, the use of population‐averaged AIFs is considered valid in clinical settings because it is time‐saving and less operator‐dependent.^31^, ^32^

Nalepa et al.^33^ developed a fully‐automated deep‐learning‐powered system for DCE‐MRI analysis of brain tumors and proposed an end‐to‐end method integrated with brain tumor segmentation, determination of vascular input function, and PK model fitting. Their end‐to‐end method appears to be important and useful, particularly in clinical settings, and is also our goal. In this study, the venous output function was used for calibration (Equation 10) to avoid the partial volume effect on AIF. The venous output function can be easily obtained by finding the pixel with the maximum CA concentration. In addition, our method does not require any PK model fitting. These aspects may be advantageous for developing a fully automated end‐to‐end method.

## 5 CONCLUSIONS

This study quantitatively evaluated the performance of two types of RNNs (LSTM and GRU) with MCD for predicting PK parameters from DCE‐MRI data through simulation and clinical studies. Our findings demonstrated the efficacy of MCD in enhancing PK parameter prediction accuracy, reliability, and uncertainty quantification at the expense of increased computational cost. Our results may improve our understanding of RNN performance in predicting PK parameters and provide value insights for selecting hyperparameters in establishing DCE‐MRI‐based RNN architecture.

## AUTHOR CONTRIBUTIONS

Kenya Murase contributed to the conceptualization, methodology, data curation, software, original draft preparation, and writing. Atsushi Nakamoto and Noriyuki Tomiyama contributed to the revising and editing.

## CONFLICT OF INTEREST STATEMENT

The authors declare no competing interest.

## Data Availability

The data that support the findings of this study are available from the corresponding author upon reasonable request.
